# Effects of 12-Week Methylphenidate Treatment on Neurometabolism in Adult Patients with ADHD: The First Double-Blind Placebo-Controlled MR Spectroscopy Study

**DOI:** 10.3390/jcm9082601

**Published:** 2020-08-11

**Authors:** Simon Maier, Ludger Tebartz van Elst, Alexandra Philipsen, Thomas Lange, Bernd Feige, Volkmar Glauche, Kathrin Nickel, Swantje Matthies, Barbara Alm, Esther Sobanski, Katharina Domschke, Evgeniy Perlov, Dominique Endres

**Affiliations:** 1 Section for Experimental Neuropsychiatry, Department of Psychiatry and Psychotherapy, Medical Center—University of Freiburg, Faculty of Medicine, University of Freiburg, 79104 Freiburg, Germany; simon.maier@uniklinik-freiburg.de (S.M.); bernd.feige@uniklinik-freiburg.de (B.F.); kathrin.nickel@uniklinik-freiburg.de (K.N.); evgeniy.perlov@uniklinik-freiburg.de (E.P.); dominique.endres@uniklinik-freiburg.de (D.E.); 2 Department of Psychiatry and Psychotherapy, Medical Center—University of Freiburg, Faculty of Medicine, University of Freiburg, 79104 Freiburg, Germany; swantje.matthies@uniklinik-freiburg.de (S.M.); katharina.domschke@uniklinik-freiburg.de (K.D.); 3 Department of Psychiatry and Psychotherapy, University Hospital Bonn, 53105 Bonn, Germany; Alexandra.Philipsen@ukbonn.de; 4 Department of Radiology, Medical Physics, Medical Center—University of Freiburg, Faculty of Medicine, University of Freiburg, 79106 Freiburg, Germany; thomas.lange@uniklinik-freiburg.de; 5 Department of Neurology, Medical Center—University of Freiburg, Faculty of Medicine, University of Freiburg, 79106 Freiburg, Germany; volkmar.glauche@uniklinik-freiburg.de; 6 Department of Psychiatry and Psychotherapy, Central Institute of Mental Health, Medical Faculty of Mannheim, University of Heidelberg, 68159 Mannheim, Germany; Barbara.Alm@zi-mannheim.de (B.A.); e.sobanski@rfk.landeskrankenhaus.de (E.S.); 7 Department of Child and Adolescent Psychiatry, University Medical Center Mainz, 55131 Mainz, Germany; 8 Center for Basics in Neuromodulation, Faculty of Medicine, University of Freiburg, 79106 Freiburg, Germany; 9 Clinic for Psychiatry Luzern, St. Urban, 4915 Luzern, Switzerland

**Keywords:** ADHD, MR spectroscopy, MRS, methylphenidate, glutamate

## Abstract

Attention deficit hyperactivity disorder (ADHD) is a frequent neurodevelopmental disorder that often persists into adulthood. Methylphenidate (MPH) is the first-line treatment for ADHD; however, despite its wide usage, little is known about its neurometabolic effects. Until now, no randomized and blinded clinical trials have been conducted addressing the neurometabolic signals of MPH administration in adults with ADHD. In the current study, the authors investigated how MPH intake and group psychotherapy (GPT) influence brain neurometabolism over the course of three months. The authors hypothesized a decrease in the anterior cingulate cortex (ACC) glutamate concentration following MPH administration. This study was part of a double-blind multicenter trial (Comparison of Methylphenidate and Psychotherapy in Adult ADHD Study (COMPAS)) investigating the effects of MPH and GPT in patients with adult ADHD. Using single-voxel magnetic resonance spectroscopy (MRS) of the pregenual ACC and the left cerebellar hemisphere (CHL), we investigated the concentration of glutamate plus glutamine (Glx), N-acetyl-aspartate, creatine, total choline containing compounds, and myo-inositol in patients before and after 12 weeks of treatment. Neither MPH nor GPT significantly influenced the Glx concentration or any of the other metabolite concentrations in the ACC and CHL after 12 weeks. Therefore, contrary to the hypothesis, no change in the prefrontal Glx signal was detected after MPH treatment. Given that MRS does not differentiate between glutamate in the synaptic cleft and in neuronal tissue, MPH-induced down-regulation of glutamatergic neurotransmission in the ACC might only affect the concentration of glutamate in the synaptic cleft, while the general availability of glutamate in the respective neuronal tissue might be unaffected by MPH intake. The observed lack of any MPH-induced normalization in metabolite concentrations is less surprising, considering that the baseline sample did not significantly differ from a healthy control group. Future studies of other regions, such as the basal ganglia, and the use of novel methods, such as whole brain MRS and multimodal imaging approaches, are necessary.

## 1. Introduction

Attention deficit hyperactivity disorder (ADHD) is a frequent neurodevelopmental disorder mainly diagnosed in childhood or adolescence [1,2]. Despite the reduction in several symptoms with age, the persistence of some other symptoms into adulthood has brought ADHD into the focus of adult psychiatry [3,4,5]. Difficulties in maintaining attention, hyperactivity and impulsiveness are central symptoms of ADHD in childhood. In about 50% of patients, these symptoms disappear when they reach adulthood, so that the diagnostic criteria are no longer met [6,7]. In patients who are still seriously affected in adulthood, hyperactivity is often reduced, whereas attention deficits, emotional instability, and executive dysfunction persist and can lead to comorbid disorders such as anxiety, depression, and alcohol or drug abuse [1,8,9]. The prevalence rates in adulthood range from 1.4% to 3.6% [10]. Multimodal treatment includes stimulant medication with methylphenidate (MPH), but also psychotherapy and psychosocial interventions [2,7,11,12].

Magnetic resonance spectroscopy (MRS) is a unique non-invasive and non-radiative method used for assessing the neurometabolism of the human brain in vivo [13,14]. In contrast to positron emission tomography (PET) and other approaches involving ionizing radiation, MRS is presumably free of side effects and, thus, is a suitable tool for longitudinal investigations. Standard single-voxel (SVS) proton MRS allows the detection of N-acetyl-aspartate (NAA), a marker of neuronal integrity; creatine (Cre), a metabolic parameter; total choline-containing compounds (t-Cho), a marker for cell connectivity; myo-inositol (mI), an important second messenger; and glutamate (Glu) and glutamine (Gln). Glu is the most important excitatory neurotransmitter and Glu + Gln are also markers for metabolism (since the transformation from Gln to Glu is an energy dependent process). Glu and Gln are often reported in combination as Glx [13,14]. Unfortunately, dopamine and norepinephrine—which are both thought to play key roles in ADHD pathogenesis—are not measurable via MRS due to their low concentrations.

Most previous MRS studies on ADHD have focused on children and adolescents, and studies addressing treatment-related metabolic changes in ADHD are rare in adults [15,16,17,18,19,20,21,22]. A literature review of the available studies using MRS to assess the effect of stimulant treatment is presented in Table 1. No placebo (Plac)-controlled study has been published to date. Available MRS data show a shift in concentrations of NAA, Cre, t-Cho, and Glu in patients after treatment with MPH. PET studies showed an association of ADHD with reduced dopamine transporter and D2/D3 receptor availability and an increase in dopamine neurotransmission after the administration of MPH [23,24,25]. Taken together with the well-known reciprocal influence of dopamine on Glu [26], these findings point indirectly to glutamatergic disturbances in ADHD. Altered levels of Glu and its normalization after treatment with MPH were reported earlier [15,16,27,28].

In the MRS part of the “Comparison of Methylphenidate and Psychotherapy in Adult ADHD Study” (COMPAS) we investigated neurometabolite concentrations in the pregenual anterior cingulate cortex (ACC) and left cerebellar hemisphere (CHL). The ACC is part of the fronto-striato-thalamo-frontal circuits that are suspected of being impaired in ADHD [6,19]. These circuits originate in the ACC (and other prefrontal areas), projecting via the striatum and the pallidum/substantia nigra to the ventrolateral thalamus and finally closing the loop by reentering the original prefrontal cortical brain areas [37,38,39]. In modulating the different fronto-striato-thalomo-reentrant circuits the mesolimbic dopaminergic system—which closely interacts with the glutamatergic system—is likely to modulate the different symptoms of ADHD [19,26,40,41]. Glu is serving directly as an accelerator or indirectly over the γ-aminobutyric acid (GABA)-ergic neurons as an inhibitor of dopaminergic neurotransmission [26]. We also investigated the cerebellum, since it is well connected to the fronto-striatal circuits over the thalamus [42]. In both regions, we detected glutamatergic alterations in our previous cross-sectional MRS studies [40,41].

### Rationale

The COMPAS-MRS study addressed two major goals. First, it evaluated cross-sectional differences in the neurochemical profile between ADHD patients and controls in ACC and the CHL. In contrast to earlier studies, this cross-sectional analyses showed no ADHD-related group difference in the MRS signal [43]. Second, it investigated how MPH—in contrast to Plac—influences the neurometabolic profile in the ACC and the CHL in a double-blind placebo-controlled prospective study in adults with ADHD. Based on previous evidence (Table 1), we initially hypothesized a shift toward normalization in NAA, Glu, Cre, and t-Cho neurometabolism as a response to MPH treatment. However, after the cross-sectional study revealed no metabolic differences in the largest adult ADHD sample studied to date [43] we could no longer hypothesize a normalization of NAA, Glu, Cre, and t-Cho. Still, we believed the Glu signal would decrease in response to an MPH upregulation of the ACC. For other metabolites, we expected no MPH-dependent change.

## 2. Participants and Methods

This study was part of the multicenter COMPAS trial and was approved by the ethical committee (Faculty of Medicine, Freiburg University, 217/06) and the German authorities for pharmacological trials (EudraCT No.: 2006-000222-31). The study was performed in accordance with the Declaration of Helsinki. The trial was registered before it began by Current Controlled Trials (ISRCTN54096201). The study protocol is available on the internet. The procedure concerning the clinical trial and the sample acquisition for the spectroscopic cross-sectional analysis was already published [43,44,45]. All participants gave their written informed consent to participate in the repeated magnetic resonance (MR) examinations.

### 2.1. Patient Recruitment and Assessment

In the double-blind multicenter COMPAS trial, a four-arm design was used to investigate the effect of MPH treatment versus a Plac and group psychotherapy (GPT) versus clinical management (CM) in adults with ADHD [44,46]. The diagnostic procedure followed the protocol of the COMPAS trial [46]. After pre-screening 1480 patients, 518 were considered for trial participation in all seven study centers. A total of 433 patients were randomly assigned into either the GPT + MPH, GPT + Plac, CM + MPH or CM + Plac arms of the study. All participants were free of stimulant medication and any substance abuse for at least six months prior to the first scan. The eligibility criteria and diagnostic instruments used in the study were published earlier [43,44,45]. For the MRS study, only patients from the Freiburg and Mannheim study centers were recruited. Therefore, all imaging data could be acquired with the same magnetic resonance imaging (MRI) scanner in Freiburg to reduce the variance in the MRS signal. The MRS measurements and psychometric assessments were performed at baseline (after the diagnostic procedure and prior to the beginning of treatment) and 12 weeks after the treatment started (in week 13).

### 2.2. Randomization and Masking

Patients eligible for study participation were randomly assigned to batches of 14 to 15. However, on one occasion, 12 patients were assigned into a single batch, and 16 patients were assigned on another occasion. The randomization parameters allowed GPT groups ranging from 6 to 9 patients. CM and GPT plus a medication number (either MPH or Plac) were centrally assigned to each patient. Treatments were allocated with a 1:1:1:1 ratio, which was stratified by center. Both patients and therapists were blinded for medication but were aware of the psychotherapy treatment (GPT or CM). MPH or Plac medication were prescribed with flexible doses. The drug was administered in capsules each containing 10 mg methylphenidate hydrochloride or in corresponding Plac capsules. Both MPH and Plac contained sucrose, gelatin, corn starch, methacrylic acid-ethyl acrylate copolymer (1:1), talc, triethyl citrate, titanium dioxide, polysorbate 80, sodium hydroxide, sodium dodecyl sulphate, simeticone, highly dispersed silica, poly(vinyl) alcohol and macrogol 3350, indigocarmine, aluminum salt, erythrosine, methyl cellulose, sorbic acid, and purified water. Carbohydrates per capsule were 10 mg (MPH) and 20 mg (Placebo), respectively. MPH capsules additionally contained 10 mg methylphenidate hydrochloride (Medikinet^®^ retard 10 mg, MEDICE Arzneimittel Pütter GmbH & Co KG, Iserlohn, Germany). The “dose” of the placebo is the MPH equivalent dose that the participant would have taken with the same amount of MPH capsules. The raters of ADHD symptoms according to the Conner’s adult ADHD rating scale (CAARS) and the checklist for diagnosis for ADHD in adults were blinded to the allocation of medication and psychotherapy [44].

### 2.3. Sample Definition

#### 2.3.1. Baseline Sample (Week 0)

All patients who passed the pre-screening from the study centers in Freiburg and Mannheim were invited to participate in the MRS study. However, at this stage, the screening process was not yet completed, and patients could still be excluded from randomization if they failed to fulfill the study eligibility criteria. Of the 187 patients who agreed to participate in the MRS study, 113 patients with ACC spectra and 104 patients with CHL spectra could be included in the MRS cross-sectional study at week 0 [43].

#### 2.3.2. Three-Month Sample (After 12 Weeks)

Of the 113 patients with ACC spectra included in the baseline sample at week 0, we were able to obtain high-quality spectra of 73 patients at week 13. Of the 104 eligible CHL spectra at baseline, we were able to include 62 into the three-month sample.

### 2.4. Data Acquisition and Analysis

**Figure 1:**
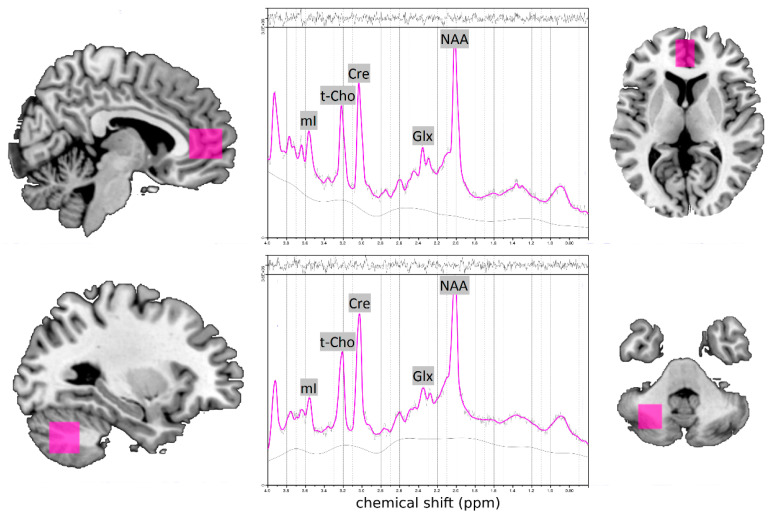
Voxel localization for MRS measurements in the pregenual anterior cingulate cortex (ACC; top left and right) and in the left cerebellum (CHL; bottom left and right) and illustrative spectra from the ACC and CHL of participants (in the middle) (adopted by Endres et al. [48]). Abbreviations: Cre,  creatine; Glx, glutamate  +  glutamine; mI, myo-inositol; NAA, N-acetylaspartate, ppm, parts per million, t-Cho, total choline compounds including phosphorylcholine  +  glycerylphosphorylcholine.

All measurements were performed at the University Medical Center Freiburg using a 3 Tesla whole body scanner (Siemens, TIM Trio System; Erlangen, Germany) with a 12-channel head coil. The data acquisition followed the same established protocol at both time points (week 0 and week 13) [43,47,48]. The morphological T1-weighted magnetization-prepared rapid-acquisition gradient echo (field-of-view = 256 mm × 256 mm, repetition time (TR) = 2200 ms, echo time (TE) = 4.11 ms, flip angle = 12°, and voxel size = 1 mm × 1 mm × 1 mm) images were obtained and used for manual localization of spectroscopic voxels in the pregenual ACC (16 × 25 × 20 mm) and in the CHL (20 × 20 × 20 mm; Figure 1). The authors were not able to reach a reasonable quality of MR spectra in the initially targeted striatal region of interest. After initial automated adjustments, the authors readjusted the shimming parameters manually to minimize the full width at half maximum of the water resonance in the region of interest. A point resolved spectroscopy (PRESS) sequence with a TE of 30 ms, a TR of 3000 ms, 256 averages, and water saturation was used. A water-reference spectrum was obtained using 16 averages of the same PRESS sequence without water saturation [49]. The well-established linear combination of the model spectra (LCModel) algorithm was used for spectral analysis [50,51], using metabolite basis spectra acquired from a phantom solution. The absolute metabolite concentrations of Cre, NAA, t-Cho, Glx, and mI were estimated with the internal water reference method [49,50,51]. Only spectra with Cramér-Rao lower bounds for the main metabolites below 20% were included in the analyses (http://s-provencher.com/lcm-manual.shtml; accessed on 23 May 2020). The measured metabolite concentrations were corrected for T1 and T2 relaxation with relaxation constants obtained from the literature. All voxels were segmented into gray matter, white matter, and cerebrospinal fluid according to the co-registered voxel position of the corresponding morphological T1-weighted image using Statistical Parametric Mapping, version 8 (London, UK; [52]).

### 2.5. Statistical Analyses

Statistical analyses were performed using the Statistical Package for the Social Sciences, version 24 (IBM Corp., Armonk, NY, USA). Since we always performed two repeated measures multivariate analysis of covariance (MANCOVA) for ACC and CHL, we put the level of significance at *p* = 0.025 following the Bonferroni correction for multiple comparisons.

#### 2.5.1. Comparison of Psychometric and Demographic Data

The MPH and Plac cohort were compared for the 13-week-sample, applying *χ^2^* tests for categorical variables and two-sample t-tests for scalar variables.

#### 2.5.2. Influence of MPH (and Psychotherapy)

To test whether MPH (or GPT) had an influence on the MR spectra, we calculated a repeated measure MANCOVA with metabolite concentrations as dependent variables, time point as the within-subject factor, medication (MPH or Plac), and psychotherapy (GPT or CM) as between-subject fixed factors. We reported the interaction of medication by time point and psychotherapy by time point, as well as the interaction of medication and psychotherapy.

## 3. Results

### 3.1. Demographic and Psychometric Findings

Both cohorts, MPH and Plac, were comparable with respect to age, sex, IQ, CAARS, and ADHD symptoms at childhood, according to the Wender Utah Rating Scale-German short version (WURS-k). As expected, the final dose of medication (MPH vs. Plac) was significantly higher in the Plac group (Table 2).

### 3.2. Longitudinal Analysis of Metabolite Concentrations

There was no interaction of visit (week 0 to week 13) by medication (MPH vs. Plac) in NAA, Glx, t-Cho, Cre, and mI levels in the ACC and the CHL (Table 3).

### 3.3. Influence of MPH and Psychotherapy

Neither the factor medication nor psychotherapy had a significant effect on metabolite concentrations after 12 weeks. The Wilks’ lambda for the effect of MPH was *F* = 1.07; *p* = 0.383; χ^2^ = 0.076 and *F* = 0.50; *p* = 0.775; χ^2^ = 0.44, for the effect of GPT it was *F* = 0.79; *p* = 0.563; χ^2^ = 0.057 and *F* = 0.21; *p* = 0.958; χ^2^ = 0.019, and for the interaction of MPH and GPT effects it was *F* = 0.94; *p* = 0.464; χ^2^ = 0.067 and *F* = 1.18; *p* = 0.334; χ^2^ = 0.098, for ACC and CHL, respectively.

## 4. Discussion

This is the first longitudinal double-blind placebo-controlled study on MPH and GPT focusing on Glx and other neurometabolites that have been studied using MRS. The main finding of this study is a lack of significant changes in neurometabolic concentrations between the MPH and Plac groups. Our initial hypothesis of normalization in the NAA, Glu, and t-Cho concentrations in response to MPH administration could not be confirmed. Yet, since those signals were already unaltered in the baseline sample [43], the absence of metabolic changes following MPH intake over a period of three months was expected. More surprising was the lack of any changes in the Glx signal since we had expected a decrease in the Glx concentration in response to MPH intake. Furthermore, there was no evidence of any significant changes in metabolite concentration following GPT.

### 4.1. Relevance of the Main Findings

The absence of any significant change in metabolite concentration in the MPH cohort after 12 weeks of MPH intake with an average dose of 0.64 mg/kg is surprising, especially for the Glx signal in the ACC. Our a priori hypothesis was based on the assumption of a disturbed Glx–dopamine interaction as a basis for ADHD pathophysiology. Following the model of dopaminergic–glutamatergic interaction in fronto-basal circuits [26], the increase in synaptic dopamine concentration in the ventral tegmentum, striatum, and other parts of the mesolimbic dopaminergic system after administration of MPH would be expected to result in a decrease in glutamatergic neurotransmission in frontal cortical areas, especially in the ACC, which is thought to be responsible for monitoring behavior and attention. We also analyzed glutamatergic changes in the cerebellum, since this region is well connected to the fronto-striatal circuits and serves as an internal modeling unit for motor and non-motor behavioral comparison and smoothing [42]. This presumed Glx decrease in the ACC was also based on earlier findings of Glx signal decline after MPH intake in smaller studies [28,32,40]. Although our study cannot confirm these assumptions, it should be noted that MRS cannot differentiate between Glu in the synaptic cleft and in neuronal tissue. Therefore, MPH-induced down-regulation of glutamatergic neurotransmission in the ACC could only affect concentration of Glu in the synaptic cleft, while the general availability of Glu in the respective neuronal tissue might be unaffected by MPH intake. Volkow et al. [53] framed a pharmacodynamic model of MPH, predicting a decrease in the background-firing rate of dopamine-norepinephrinergic neurons after psychostimulant administration, which, in turn, would lead to an increase in the signal-to-noise ratio, resulting in an optimization of the task-dependent glucose metabolism. Following the model of Tod and Botteron [54], optimization in glucose metabolism is a key mechanism of action for MPH, since, according to this model, ADHD reflects a state of energy deficiency. Since glutamatergic neurotransmission is strictly energy dependent, we expected to observe changes in the Glx signal as a consequence of MPH intake. Following this line of thought, one could also expect changes in the Cre signal, since Cre is a marker for energy metabolism. Although changes in the Cre concentration would possibly affect energy metabolism, changes in energy metabolism are not necessarily reflected by a change in Cre signal, since Cre functions as a buffer in Adenosine 5′-triphosphate (ATP) turnover, leading to a shift in Cre/phosphocreatine equilibrium when ATP is hydrolyzed to adenosine 5′-diphosphate. This shift in the Cre/phosphocreatine ratio cannot be detected in the Cre MR spectrum-peak, since the MRS peaks of both metabolites are superimposed. To separate the two signals in future studies, the use of phosphorus MRS could be applied.

### 4.2. Link to Previous Findings

The hypotheses of MPH-induced changes in MRS metabolite concentrations could not be confirmed. In general, the findings of earlier MRS studies addressing MPH effects or metabolic differences of children and adults with ADHD could not be consistently confirmed in later studies (Table 1). Elevated Glu or Glx have been reported in response to therapy with stimulants in the striatum [17], contrasting reports of decreased Glx in the striatum [28]. While Glx is reportedly elevated in the left anterior centrum semiovale [34], it is lowered in the prefrontal cortex (PFC) and the amygdala following stimulant medication [28,32,40]. In line with our study, several other studies also failed to show stimulant dependent changes in the Glx signal in the striatum, the ACC, the prefrontal cortex (PFC), or the right centrum semiovale in children and adults [17,31,36]. Changes in the NAA signal were also reported in several studies following treatment with MPH; however, these results were also inconsistent (Table 1). Jin et al. [29] and Husarova et al. [34] reported a decrease in NAA concentrations after treatment; Jin et al. [29] in the striatum, Husarova et al. [34] in the left dorsolateral PFC. Meanwhile, Kronenberg et al. [30] showed an increase in NAA in the ACC in adults. All other studies revealed no changes in NAA concentrations related to stimulant treatment (Table 1). Reports of changes in Cre concentrations are rare, presumably due to the fact that Cre is commonly used as a divisor in metabolite ratios. One study showed a decrease in Cre as a single metabolite after treatment with MPH [17]; another study discerned a cerebellar Cre increase in adults after MPH use [35]. The t-Cho was reported to be elevated after treatment in the striatum [29] and in the right DLPFC [34]. However, it has been shown to decrease in ACC in adults [30] and in PFC in children [32] after treatment. After MPH treatment, early-stimulant-treated patients showed an increase in GABA levels [36]. Further studies investigating changes in the GABA signal after MPH treatment are still pending. The differences in the power of studies (some of which are only case series), designs, methodical aspects in the MRS technique, MPH dosages, duration of therapy (varying from a single dose to several weeks of regular treatment), and the regions investigated make it difficult to compare the results. Most studies that reported changes in Glu signal in response to MPH intake measured short-term effects and thus might measure a temporary adjustment of the neurotransmission to higher levels of dopamine and noradrenaline during MPH admission. To our knowledge, there have been no studies addressing the duration of the MPH effect on the Glx signal, and it is unclear how the Glx signal adapts to MPH over time. Supposedly, the first adaptive response involving changes in metabolite concentrations is later replaced by longer-lasting changes in activation, structure, connectivity, or receptor density [55,56,57]. The inconsistency of the findings across different brain regions, but partly also in identical brain regions, suggests that possible changes in metabolite concentration related to MPH treatment are mainly local rather than global.

Due to the very limited evidence of psychotherapeutic effects on neurometabolite concentrations, the authors refrained from building any a priori hypothesis related to psychotherapeutic intervention. To our knowledge, this is the first study addressing this issue in ADHD. There are a few MRS studies on the effect of psychotherapy in other disorders, such as major depression or obsessive-compulsive disorder. Abdallah et al. [58] showed a decrease in Glu in the occipital cortex depending on the response to cognitive behavioral therapy (CBT), but they did not observe changes in GABA neurotransmission according to their primary hypothesis. In a study investigating the effect of CBT in obsessive-compulsive patients, an increase in NAA and decrease in Glx were described in different parts of the ACC [59]. A study investigating spectroscopic changes after Zen meditation is also interesting in relation to the results of our study since some Zen Buddhistic elements, such as attentiveness, were integrated in our therapeutic GPT procedure. Fayed at al. [60] were able to show changes in mI, NAA, and Glu in the ACC and the thalamus of meditators correlating with years of practice. In summary, it seems too early to draw conclusions concerning the effects of psychotherapy on neurometabolite concentrations. There is still a lot of work to do, and our study, which shows no changes in the neurometabolism after three months of therapy, is only another brick in the wall. Possibly, the effects of psychotherapy are too subtle to be consistently detected with the currently used MRS methodology.

### 4.3. Limitations

Although the amount of medication prescribed and the psychotherapy arms of our sample were highly comparable to previous trials, a much higher dropout rate than anticipated resulted in a smaller sample size than expected; even so, this study is still the largest study to date investigating the effects of MPH using MRS. The reasons for the high dropout rate were probably related to the expenditure of time for participation in the therapy program, the disorder itself, and the fact that subjects could only be included when the MRS measures at both time points could be obtained and passed quality controls. In particular, movement-related artifacts and compliance/adherence to the appointed scanning dates were issues in the ADHD population. The effect of MPH on metabolite concentration, if it exists, might be too subtle to be detected in 3 T 1H-MRS with the studied sample size. Apart from the limited number of detectable metabolites in MRS, important changes in the dynamics or exact localization (synaptic cleft versus tissue) of the measurable metabolites could not be assessed as well. As mentioned before, MPH-induced changes in energy metabolism presumably affect the creatine–phosphocreatine equilibrium without changing the recorded Cre peak consisting of both metabolites. Hence, it is important to keep in mind that a lack of signal change in the MRS spectra does not rule out the possibility of changes in the dynamics of different neurometabolites. The authors were not able to reach a reasonable quality of MR spectra in the initially targeted striatal region of interest. This region was therefore not investigated as initially planned.

## 5. Conclusions

The hypothesized changes in metabolic concentrations in the ACC could not be proven in our study. This finding is not surprising since we hypothesized the normalization of metabolic concentrations in ADHD after treatment without having detected these changes in the same group before treatment. The most surprising fact is the lack of a decrease in the Glx signal after MPH administration, which was assumed based on Carlsson et al.’s [26] model. Keeping in mind that this study could only make statements about the average metabolic concentration and combinations of metabolites in two relatively large regions of brain tissue, we could have missed the crucial regions of interest if the changes were local and not global. Further studies in basal ganglia, especially the striatum, or even with newer methodologies, such as whole brain MRS (which is becoming increasingly established) and multimodal approaches, could be worthwhile in future interventional ADHD research.

## Figures and Tables

**Table 1:** Summary of previous magnetic resonance spectroscopy studies analyzing medication effects in patients with ADHD.

Study	Population	*n* (ADHD/ Contr.)	Methods	Region(s)	Results
1. Jin et al., 2001 [29]	Children	12/10	1,9 T, SVS, 1H-MRS (PRESS); MPH once	Striatum le	NAA/Cre ↓
				Striatum ri	NAA/Cre ↓, t-Cho/Cre ↑; MPH-once without effect
2. Carrey et al., 2002 [28]	Children	4/0	1,5 T, SVS, 1H-MRS (PRESS); before and after MPH/AM	Striatum le	Glu/Cre ↓ under MPH + AM
				PFC ri	Glu/Cre ↓only under AM
3. Carrey et al., 2003 [16]	Children; iADHD, cADHD	14/0	1,5 T, SVS, 1H-MRS (PRESS); before and after MPH (4)/AM (3)/Dexedrine (7)	Striatum le	Glx/Cre ↓ under medication
				PFC ri	↔
4. Carrey et al., 2007 [17]	Children; cADHD	13/10	1,5 T, SVS, 1H-MRS (PRESS); before and after MPH	Striatum le	Glx ↑, Cre ↑; Cre ↓ after MPH
				PFC ri	↔
				Occipital lobe	↔
5. Kronenberg et al., 2008 [30]	Adults	7/0	1,5 T, CSI, 1H-MRS (PRESS); before and after MPH	ACC both sides	t-Cho↓, NAA↑ after MPH, Cre ↔
6. Hammerness et al., 2010 [31]	Children	10/12	4 T, SVS, 1H-MRS (PRESS); before and after MPH	ACC both sides	↔, no significant changes under MPH
7. Wiguna et al., 2012 [32]	Children; iADHD, cADHD	21/0	1,5 T, SVS, 1H-MRS; before and after MPH	PFC le	NAA/Cre ↑, Glu/Cre ↓, t-Cho/Cre ↓, mI/Cr ↓ after MPH
				PFC ri	NAA/Cre ↑, Glu/Cre ↓, t-Cho/Cre ↓ after MPH
8. Wiguna et al., 2014 [33]	Children; cADHD and iADHD	21/0	1,5 T, SVS, 1H-MRS; before and after MPH	Amygdala le	Glu/Cre ↓ after MPH
				Amygdala ri	Glu/Cre ↓ after MPH
9. Husarova et al., 2014 [34]	Children; cADHD	21/0	1,5 T, SVS, 1H-MRS (PRESS); before and after MPH/AM	DLPFC le	NAA and NAA/Cre ↓ after AM
				DLPFC ri	t-Cho/Cre ↑ after AM
				Anterior semioval center le	Glx ↑ and Glx/Cre ↑ after MPH
				Anterior semioval center ri	↔
10. Inci Kenar et al., 2017 [35]	Adults	60/0	1,5 T, SVS, 1H-MRS (PRESS); before and after MPH	DLPFC	↔ after MPH
				Striatum	↔ after MPH
				ACC	↔ after MPH
				Cerebellum	Cre ↑ after MPH ↑
11. Solleveld et al., 2017 [36]	Adult (early-stimulant-treated (<16 years) vs. late-stimulant-treated (>23 years) vs. stimulant-treatment-naive patients)	44/0	3 T, SVS, 1H-MRS (PRESS); before and after MPH	mPFC both sides	GABA lower in early-stimulant-treated vs. late-stimulant-treated. After MPH only the early-stimulant-treated patients showed increase in GABA. No Glx differences at baseline or after MPH.

Abbreviations: ADHD, attention deficit hyperactivity disorder; Cre, creatine; t-Cho, phosphorylcholine + glycerylphosphorylcholine; GABA, γ-aminobutyric acid; Glu, glutamate; Glx, glutamate + glutamine; NAA, N-acetylaspartate; mI, myo-Inositol; cont., controls; le, left; ri, right; ↑, increased in ADHD; ↓, decreased in ADHD; ↔, no metabolite differences between groups; MPH, methylphenidate; AM, atomoxetine; iADHD, inattentive ADHD subtype; cADHD, combined ADHD subtype; MRS, magnetic resonance spectroscopy; PRESS, point-resolved spectroscopy; T, Tesla; SVS, single-voxel spectroscopy; CSI, chemical shift imaging; 1H-MRS, proton magnetic resonance spectroscopy; DLPFC, dorsolateral prefrontal cortex; ACC, anterior cingulate cortex; PFC, prefrontal cortex.

**Table 2:** Comparison of demographic and psychometric data of methylphenidate (MPH) versus placebo (Plac) cohorts in the anterior cingulate cortex (ACC) and left cerebellar (CHL) sample.

Variable	MPH		Plac		Total		Statistics (MPH vs. Plac)	
ACC								
	***n***		***n***		***n***		**Chi^2^**	***p-*** **value**
Total	40		33		73			
Female:Male	20:20		16:17		36:37		0.02	0.897
Psychotherapy (GPT:CM)	17:23		15:18		32:41		0.06	0.800
	**Mean**	**SD**	**Mean**	**SD**	**Mean**	**SD**	***t-*** **value**	***p-*** **value**
IQ	115.05	16.3	112.67	16.3	113.97	16.2	*t*(71) = 0.62	0.536
Age (years)	34.80	10.7	35.58	9.1	35.15	10.0	*t*(71) = −0.33	0.744
WURS−k	39.78	9.5	41.88	7.2	40.73	8.5	*t*(71) = −1.05	0.298
CAARS (baseline)	107.11	30.5	105.59	34.0	106.43	31.9	*t*(71) = 0.20	0.841
BDI (baseline)	12.37	7.0	11.73	8.9	12.07	7.9	*t*(71) = 0.35	0.731
Dose (mg/kg)	0.64	0.3	0.84	0.3	0.73	0.3	*t*(71) = −3.19	0.002 *
Dose (mg)	46.38	18.8	64.09	21.1	54.38	21.7	*t*(71) = −3.79	<0.001 *
			CHL					
	***n***		***n***		***n***		**Chi^2^**	***p−*** **value**
Total	33 *n*		29		62			
Female:Male	18:15		15:14		33:29		0.049	0.824
Psychotherapy (GPT:CM)	13:20		13:16		26:36		0.187	0.665
	**Mean**	**SD**	**Mean**	**SD**	**Mean**	**SD**	***t-*** **value**	***p-*** **value**
IQ	114.21	16.7	112.59	15.8	113.45	16.2	*t*(60) = 0.39	0.696
Age (years)	34.73	11.36	35.38	9.2	35.03	10.3	*t*(60) = −0.25	0.807
WURS−k	40.67	10.1	41.28	7.5	40.95	8.9	*t*(60) = −0.27	0.790
CAARS	103.82	34.52	104.01	33.9	103.91	33.9	*t*(60) = −0.21	0.983
BDI (baseline)	12.15	7.0	11.28	8.9	11.74	7.9	*t*(60) = 0.43	0.669
Dose (mg/kg) ^†^	0.64	0.2	0.83	0.3	0.73	0.3	*t*(60) = −2.75	0.008 *
Dose (mg) ^†^	45.91	18.1	62.59	22.1	53.71	21.6	*t*(60) = −3.27	0.002 *

Abbreviation: MPH, methylphenidate; Plac, placebo; ACC, anterior cingulate cortex; CHL, left cerebral cortex; SD, standard deviation; GPT, group psychotherapy; CM, clinical management; WURS-k, Wender Utah Rating Scale; BDI, Beck Depression Inventory; CAARS, Conner’s Adult ADHD Rating Scale. ^†^ The “dose” of the placebo is the methylphenidate equivalent dose that the participant would have taken with the same amount of methylphenidate capsules. * Significant difference.

**Table 3:** The effect of methylphenidate compared with placebo at baseline and 12 weeks after therapy start.

ACC				
		T0 Metabolite concentration (Mean ± SD)	T1 Metabolite concentration (Mean ± SD)	Visit (week 0 to week 13) x Medication
**NAA**	MPH (*n* = 40)	5.60 ± 0.67	5.35 ± 0.79	F(1, 71) = 0.23; *p* = 0.632
	Placebo (*n* = 33)	5.73 ± 0.61	5.39 ± 0.59	
**Glx**	MPH (*n* = 40)	8.32 ± 1.08	7.44 ± 1.32	F(1, 71) = 0.00; *p* = 0.984
	Placebo (*n* = 33)	8.29 ± 1.10	7.43 ± 1.06	
**t-Cho**	MPH (*n* = 40)	1.12 ± 0.21	1.06 ± 0.23	F(1, 71) = 0.18; *p* = 0.671
	Placebo (*n* = 33)	1.13 ± 0.18	1.10 ± 0.16	
**Cre**	MPH (*n* = 40)	4.77 ± 0.69	4.53 ± 0.90	F(1, 71) = 0.00; *p* = 0.967
	Placebo (*n* = 33)	4.91 ± 0.69	4.67 ± 0.68	
**mI**	MPH (*n* = 40)	3.04 ± 0.48	3.04 ± 0.57	F(1, 71) = 1.35; *p* = 0.249
	Placebo (*n* = 33)	3.32 ± 0.43	3.16 ± 0.43	
**CHL**				
		**T0** **Metabolite concentration** **(Mean ± SD)**	**T1** **Metabolite concentration** **(Mean ± SD)**	**Visit (week 0 to week 13) x Medication**
**NAA**	MPH (*n* = 33)	4.95 ± 0.56	4.87 ± 0.42	F(1, 60) = 0.23; *p* = 0.631
	Placebo (*n* = 29)	4.95 ± 0.73	4.80 ± 0.36	
**Glx**	MPH (*n* = 33)	5.97 ± 1.05	5.98 ± 0.74	F(1, 60) = 0.01; *p* = 0.910
	Placebo (*n* = 29)	6.11 ± 0.94	6.08 ± 0.88	
**t-Cho**	MPH (*n* = 33)	1.31 ± 0.20	1.25 ± 0.18	F(1, 60) = 0.24; *p* = 0.627
	Placebo (*n* = 29)	1.27 ± 0.15	1.23 ± 0.11	
**Cre**	MPH (*n* = 33)	5.58 ± 0.66	5.56 ± 0.71	F(1, 60) = 0.17; *p* = 0.678
	Placebo (*n* = 29)	5.63 ± 0.46	5.54 ± 0.47	
**mI**	MPH (*n* = 33)	2.60 ± 0.56	2.86 ± 0.38	F(1, 60) = 0.14; *p* = 0.713
	Placebo (*n* = 29)	2.73 ± 0.56	2.93 ± 0.42	

Abbreviations: SD, standard deviation; NAA, N-acetylaspartate; Glx, glutamate + glutamine; t-Cho, phosphorylcholine + glycerylphosphorylcholine; Cre, creatine; mI, myo-Inositol; MPH, methylphenidate. It should be noted that the baseline concentrations measured in this work are substantially different from the concentrations reported previously [43,48]. These systematic differences are due to the fact that in contrast to earlier work where only water relaxation was corrected for with a general factor, in this work a dedicated relaxation correction based on metabolite T1 and T2 constants from the literature was performed.
